# Metabolic heterogeneity and survival outcomes in papillary renal cell carcinoma: insights from multi-datasets and machine learning analyses

**DOI:** 10.1186/s41065-025-00571-9

**Published:** 2025-09-26

**Authors:** Jian Hu, Yi-heng Liu, Gui-lian Xu, Ke-qin Zhang

**Affiliations:** 1 Urinary Nephropathy Center, The Second Affiliated Hospital, Chongqing Medical University, Chongqing, China; 2 Department of Immunology, Army Medical University (Third Military Medical University), Chongqing, China

**Keywords:** Metabolism reprograming, Renal papillary cell carcinoma, Prognosis, Machine learning

## Abstract

**Background:**

Renal cell carcinoma is characterized by immune and metabolic alterations. These metabolic reprogramming processes enhance tumor cell proliferation and infiltration. The purpose of this study was to investigate the characteristics of metabolism-related molecules and to identify potential prognostic biomarkers in kidney renal papillary renal cell carcinoma (KIRP).

**Methods:**

We conducted a comprehensive analysis of metabolism-related genes using weighted gene co-expression network analysis and differential expression analysis. Subsequently, we constructed a metabolism-related signature (MRS) by integrating 90 machine learning algorithms. Based on Cox regression analyses, we developed a predictive nomogram. Functional enrichment analysis, genomic variant analysis, chemotherapy response evaluation, and immune cell infiltration profiling were then performed among the MRS subtypes. Finally, the MRS was further examined at the single-cell level, and quantitative PCR and immunohistochemical staining were conducted to validate the key genes.

**Results:**

We identified 16 differentially expressed metabolic genes. The random survival forest (RSF) emerged as the optimal machine learning model in the TCGA-KIRP and GSE2748 cohorts. The MRS demonstrated robust predictive performance, with an AUC of 0.989 for 5-year survival predictions. The risk score was significantly correlated with T stage and pathological stage and was identified as an independent prognostic factor. Patients in the high-risk group exhibited higher tumor mutation burdens and derived greater benefits from sunitinib, pazopanib, lenvatinib, and temsirolimus. A four-genes nomogram was then constructed to predict overall survival. *PYCR1*, *INMT*, and *KIF20A* were highly expressed in KIRP according to scRNA-seq analysis and were validated in vitro.

**Conclusion:**

This study revealed the heterogeneity of metabolic molecules in KIRP and established a prognostic machine learning model that enhances risk stratification and may optimize chemotherapy strategies in the management of KIRP.

**Supplementary Information:**

The online version contains supplementary material available at 10.1186/s41065-025-00571-9.

## Introduction

Renal cell carcinoma is a common malignant solid tumor in urology, with a rising incidence worldwide [1]. Among the pathological types of renal cell carcinoma, kidney renal papillary cell carcinoma (KIRP) ranks second after clear cell renal cell carcinoma [1]. Despite advances in treatment strategies, intra-tumor heterogeneity in KIRP could potentially influence clinical treatments responses [2]. Therefore, exploring the molecular mechanisms underlying this heterogeneity and identifying reliable biomarkers are imperative for improving prognosis and therapeutic outcomes in patients with KIRP.

Metabolic alterations, also referred to as metabolic reprogramming, are intrinsic features of cancers that arise from enhanced nutrient uptake and biosynthesis to support tumor growth, progression, and metastasis [3]. Multiple studies have consistently shown that renal cell carcinoma, including KIRP, is driven by metabolic abnormalities, underscoring their crucial role in carcinogenesis and progression [3–5]. These metabolic alterations are closely associated with genetic mutations and aberrant regulation of signaling pathways. For example, the *VHL* mutations and the upregulated PI3K–AKT–mTOR signaling pathway regulate most of the metabolic reprogramming observed in renal cell carcinoma [6, 7]. *Linehan et al.* further reported that more than 17 mutated genes drive renal carcinogenesis by significantly altering cellular metabolism, including oxygen and iron metabolism [8, 9]. In KIRP, genetic variations implicated in metabolic alterations include *MET*, *FH*, *TFE3*, *TFEB*, and *MITF*, all of which foster tumor cell proliferation and survival [8]. The application of metabolomics and sequencing technologies has revealed that metabolic reprogramming in renal cell carcinoma involves not only glycolysis, fatty acid metabolism, and the tricarboxylic acid cycle but also arginine, glutamine, and tryptophan metabolism [10]. These metabolic pathways are intricate and interconnected, potentially serving as sources of intra-tumor heterogeneity [11]. Such heterogeneity may lead to variations in prognosis and treatment response. However, the metabolic molecular landscape of KIRP remains poorly defined.

In the present study, we conducted a comprehensive investigation of the molecular characteristics of metabolic reprogramming with the aim of identifying novel prognostic biomarkers and potential therapeutic strategies in KIRP.

## Materials and methods

### Data collection processing

The mRNA expression profiles, somatic mutation data, and corresponding clinical information of the KIRP cohort (*N* = 324) were obtained from The Cancer Genome Atlas (*TCGA*, https://portal.gdc.cancer.gov/) in February 2024. An external validating cohort (GSE2748, *n* = 34) were acquired from Gene Expression Omnibus (GEO, https://www.ncbi.nlm.nih.gov/) database, and the clinical characteristics were collected from the corresponding published paper [12]. After excluding samples due to incomplete data, a total of 28 patients from the GSE2748 cohort and 322 patients from the TCGA cohort were included for further analysis. Additionally, we obtained single-cell RNA sequencing (scRNA-seq) data from GSE152938, comprising one KIRP tissue and one normal renal tissue [13]. A total of 703 metabolism-related genes were *retrieved* from the Human MSigDB Collections, *encompassing key pathways such as* glycolysis, the tricarboxylic acid cycle, oxidative phosphorylation, and fatty acid metabolism.

### Weighted gene co‑expression network analysis

The Weighted Gene Co-Expression Network Analysis (WGCNA) algorithm has the ability to identify co-varying gene sets [14]. Thus, we utilized the WGCNA R package to explore co-expression gene sets and identified relationships between modules and survival time, stage, and survival state. The top 30% variable genes from the TCGA-KIRP patients were selected to construct co-expression network. The optimal soft threshold (β) was automatically calculated to achieve a scale-free topology (R² = 0.9). And then, a topological overlap matrix (TOM) and the dissimilarity (dissTOM) were then generated. Using a dynamic tree cut approach with a threshold of 0.1, modules were identified and merged. Ultimately, the module most strongly correlated with survival, survival time, and pathological stage was selected for further analysis.

### Construction of a metabolism-related signature using machine learning approaches

In the TCGA-KIRP dataset, we performed differential expression analysis of metabolism-related genes using the *limma* R package (|logFC| >1 and adjusted *P* < 0.05) [15]. The intersecting genes, identified from differentially expressed metabolism-related genes and module genes obtained through WGCNA, were used to construct a metabolism-related signature (MRS) using integrative machine learning approaches. The TCGA-KIRP dataset served as the training cohort, whereas GSE2748 was used as the validation cohort. Based on previous research, we trained 90 combinations of machine learning models by applying 9 algorithms (random survival forest [RSF], CoxBoost, SuperPC, Lasso, Ridge, stepwise Cox, survival-SVM, plsRcox, and Enet) for gene selections and prognostic model construction [16]. The concordance index (*C-index*) for each machine learning model was calculated in both the training and validation cohorts. These combinations were ranked according to their average *C-index*, and the top-ranked combinations were selected for their potential clinical relevance and superior predictive performance. A prognostic model for KIRP patients was therefore established, termed the MRS. The risk score for each patient was calculated as the weighted sum of the expression levels of the four selected genes, using the corresponding regression coefficients from the Cox model [16]. Risk scores were normalized with the *z-score* function in the *mosaic* R package. Patients with risk scores > 0 were categorized into the high-risk group, whereas those with risk scores ≤ 0 were classified into the low-risk group.

### Biological characteristic and survival analysis of MRS subtypes

Utilizing *ClusterProfiler* R package, functional enrichment analyses, including Gene Ontology (GO) and Kyoto Encyclopedia of Genes and Genomes (KEGG), were performed separately for upregulated and downregulated genes across the MRS subtypes. To assess the discriminatory ability of the MRS in risk classification, Kaplan–Meier survival curve analysis was conducted using the *survminer* R package. In addition, receiver operating characteristic (ROC) curve analysis within the *timeROC* R package was used to evaluate the sensitivity and robustness of the MRS in predicting prognosis at 1, 3, and 5 years.

### Genomic variation and immune cell infiltration

The *maftools* R package was to identify somatic mutations between the high-risk and low-risk groups, displaying the top 20 mutated genes. Using ssGSEA algorithms, we examined the landscape of 28 immune cell infiltrations across the MRS subtypes.

### Establishment and independent validation analysis of a predictive nomogram

Univariate and multivariate Cox regression analyses were performed for each hub gene within the MRS to determine whether it served as an independent risk factor for overall survival in patients with TCGA-KIRP. A nomogram incorporating all hub genes within the MRS was then constructed, with the total score predicting survival probabilities at 1, 3, and 5 years. Subsequently, calibration curves, the *C-index*, and decision curve analysis (DCA) were used to evaluate the predictive performance of the nomogram. Finally, the prognostic capability of the nomogram was independently validated in the external cohort *GSE2748*.

### Chemotherapy response assessment and scRNA-seq analysis

Using the *oncoPredict* R package, we calculated the half-maximal inhibitory concentration (IC50) of 545 chemical drugs for each TCGA-KIRP patient based on bulk RNA expression profiles [17]. The IC50 represents the drug concentration required to inhibit 50% of cell growth; a lower IC50 indicates greater drug sensitivity. We then compared the responses to tyrosine kinase inhibitors (TKIs) and mTOR inhibitors between the high-risk and low-risk groups. Spearman’s correlation analysis was applied to evaluate the relationships between the risk score and IC50 values for these drugs.

The single-cell RNA sequencing (scRNA-seq) data matrix was processed using the standard pipeline in the *Seurat* R package [18]. In the quality control section, low-quality cells—defined as those with more than 15% mitochondrial gene expression or with gene counts outside the range of 200–5000—were filtered out. The uniform manifold approximation and projection algorithm was used to visualize clustering results. Marker genes for cell type identification were obtained from previous studies to annotate clusters [13]. Furthermore, enrichment scores of the MRS in KIRP samples were quantified using the single-sample gene set enrichment analysis algorithm implemented in the *GSVA* R package [19].

### Cell culture and quantitative real‑time polymerase chain reaction (qPCR)

The human papillary renal cell carcinoma line (Caki-2, CL-0326) and the human proximal tubular epithelial cell line (HK-2, CL-0109) were purchased from Life Science & Technology. Caki-2 cells were maintained in their specific culture medium (CM-0326, Life Science & Technology), whereas HK-2 cells were cultured in their designated medium (CM-0109, Life Science & Technology). All cells were incubated at 37 °C in a humidified atmosphere containing 5% CO2.

The qPCR was conducted following the protocol from our previous study. Briefly, total RNA was extracted from Caki-2 and HK-2 cells using an RNA extraction kit (G3013, Wuhan Servicebio Technology). The extracted RNA was then reverse-transcribed into cDNA using the ABScript III RT Master Mix (RK20429, ABclonal). Real-time PCR was performed on a 96-well real-time PCR system (PCR-0802-FC, Wuhan Servicebio Technology). Relative expression levels of target genes were calculated using the comparative Ct method, with GAPDH serving as the internal control for normalization. Primer sequences used in this study are listed in Supplementary Table 1.

### Sample collection and immunohistochemical staining

Tumor tissues and matched adjacent normal tissues from three KIRP patients were obtained from the Department of Pathology, The Second Affiliated Hospital of Chongqing Medical University, with approval from the Human Research Ethics Committee *(Approval No. 446)*. Immunohistochemical staining was performed as previously described [20]. The primary antibodies used were PYCR1 (Abclonal, China, A27417), INMT (Abclonal, China, A14391), CHST2 (BIOSS, China, bs-13934R), and KIF20A (BIOSS, China, bs-7750R).

### Statistical analysis

All statistical analyses and visualizations were performed using R software (version 4.3.2). Kaplan–Meier survival curve analysis with log-rank tests, ROC curve analysis, Cox regression analyses, Wilcoxon tests, Student’s t-tests, and Spearman’s correlation tests were applied for difference and correlation analyses. Statistical significance was defined as a two-sided *P* < 0.05.

## Results

### Construction of a co-expression network and gene module selection

**Fig. 1:**
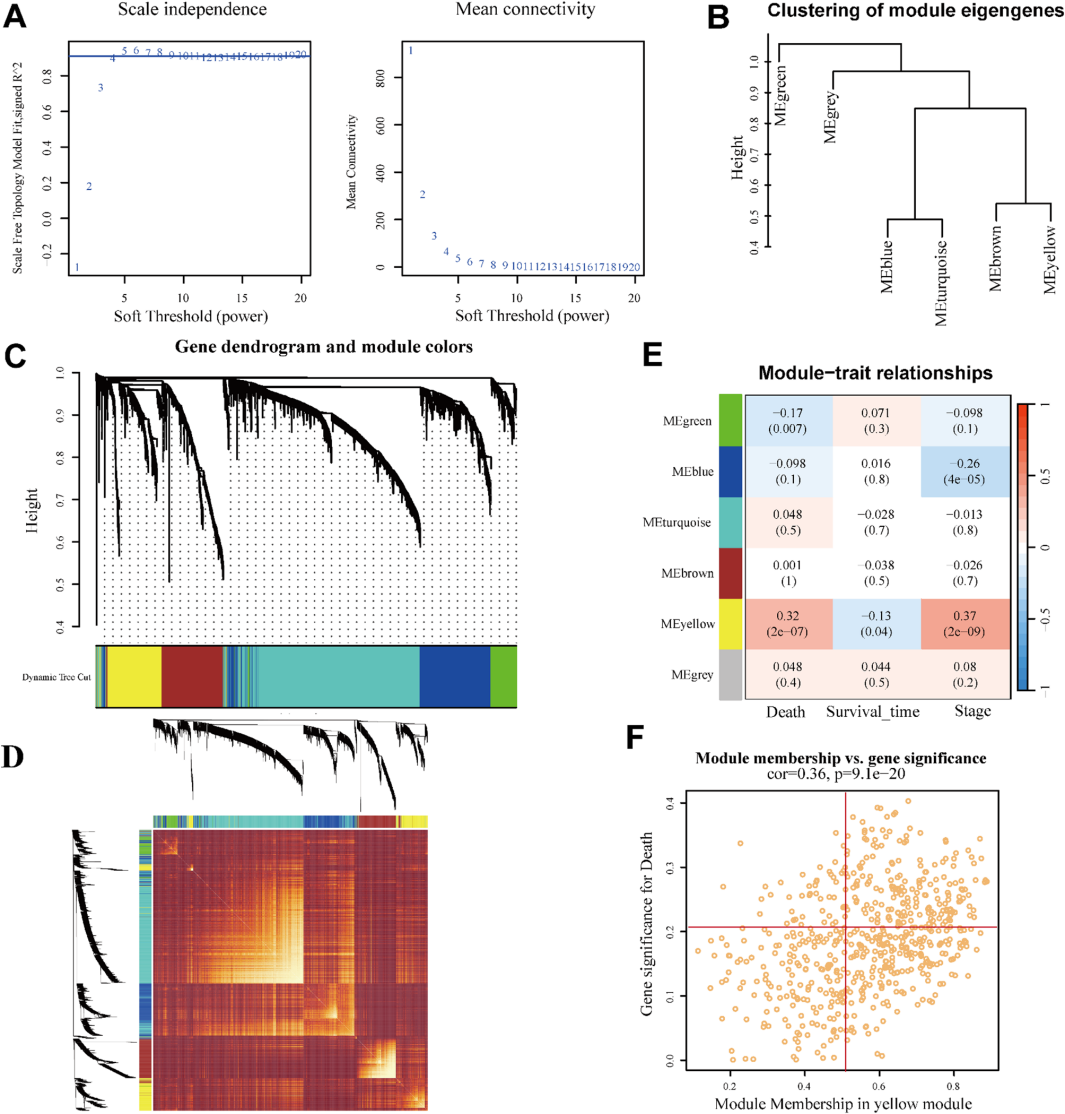
Co-expression network of prognosis in KIRP patients. (**A**) Selection of soft threshold power. (**B**) Dendrogram of the cluster tree showing co-expression modules. (**C**) Cluster diagram of module eigengenes depicted in distinct colors. (**D**) Topological overlap matrix heatmap of six modules. (**E**) Heatmap illustrating the correlation between module eigengenes and death, survival time, and pathological stage. (**F**) Scatter plot represented the genetic significance of yellow module members with respect to death

Using the WGCNA algorithm, we constructed a co-expression network for KIRP patients and identified key modules significantly associated with prognosis. The optimal soft-thresholding power (β) was set to 4, achieving an unscaled R2 value of 0.9. The top 30% of genes with the highest standard deviation were selected to build co-expression modules (Fig. 1A). Based on the dynamic cut algorithm, six independent co-expression modules were identified, and a TOM heatmap was generated (Fig. 1B and D). Among these, the MEyellow module, which contained 599 hub genes, showed the strongest correlation with KIRP stage, indicating poor prognosis (Fig. 1E). Furthermore, there was a positive correlation between the MEyellow module and death-related genes (Fig. 1F).

### Construction of the prognosis model based on integrated machine learning

**Fig. 2:**
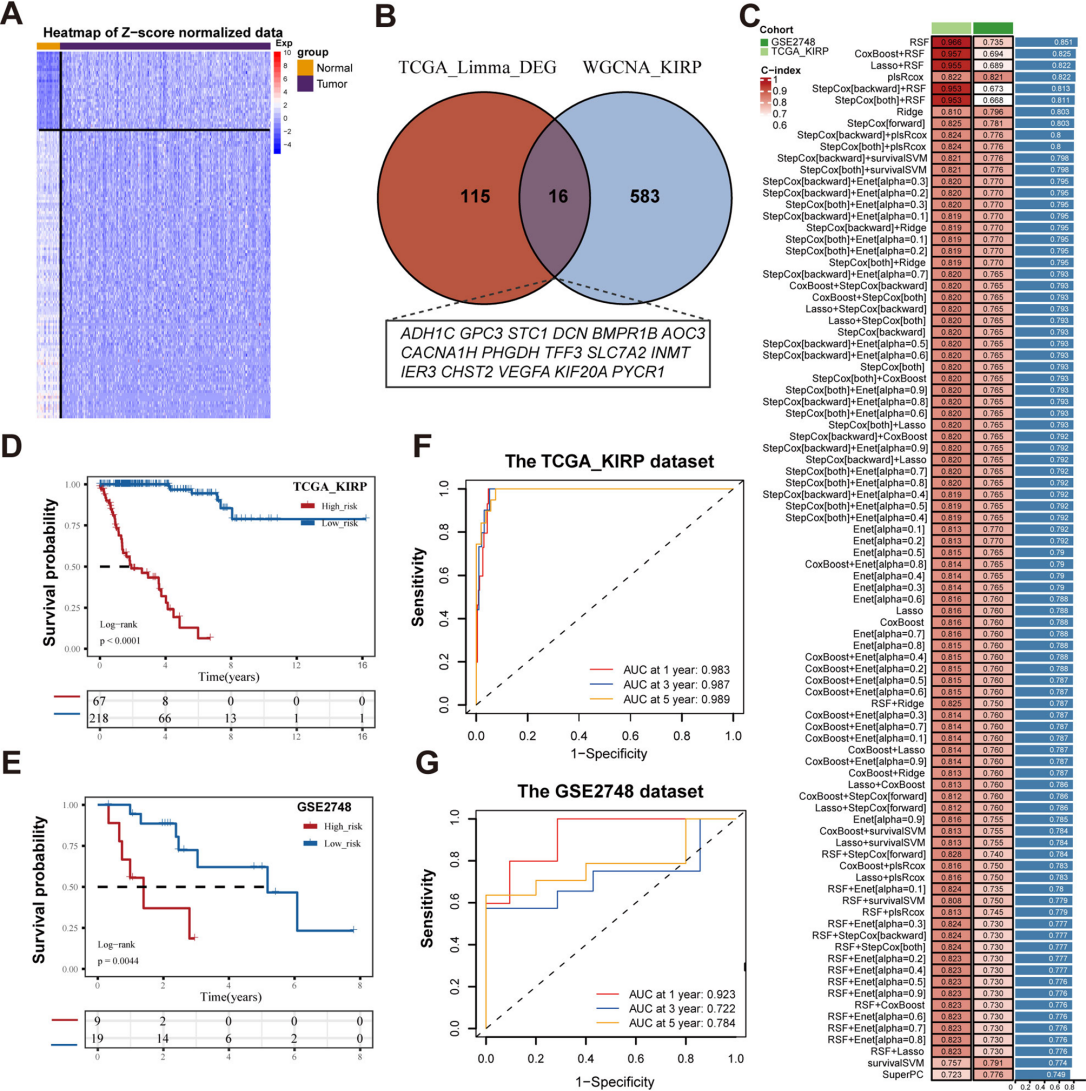
Construction of machine learning model. (**A**) The heatmap depicts the differential expression of genes between KIRP tissues and normal renal tissues. (**B**) Overlap genes between genes in the MEyellow gene module and differentially expressed genes. (**C**) 90 kinds of prediction models were developed, and the C index of each model was calculated. (**D**, **E**) Kaplan-Meier curves illustrating overall survival according to the MRS in both the TCGA-KIRP training cohort and the GSE2748 validation cohort. (**F**, **G**) ROC curves showing the specificity and sensitivity of MRS in predicting overall survival at 1, 3, and 5 years

131 differentially expressed metabolic genes were identified between normal tissues and KIRP tissues in the TCGA database, among which 28 were upregulated, while 103 were down regulated (Fig. 2A). By intersecting the differentially expressed metabolic genes and the genes from the MEyellow module associated with prognosis, a total of 16 hub genes were identified (Fig. 2B). To construct MRS, we used a combination of 90 machine-learning algorithms to analysis the above 16 genes. With a tenfold cross-validation framework, we fitted 90 prediction models in the training sets which from the TCGA-KIRP dataset, and computed the *C*-index both in the TCGA-KIRP dataset and GSE2748 dataset (Fig. 2C). Following that, the top prediction model, ranked by the average *C*-index, was the random survival forest (RSF) algorithm, which included four genes (*KIF20A*, *PYCR1*, *CHST2*, *INMT*). Compared with other machine learning models, the RSF prediction model exhibited strong performance in both the *TCGA-KIRP* dataset (*C-index = 0.966)* and *GEO2748* dataset (*C index = 0.735*). Therefore, RSF was selected as the optimal model in this study.

The predictive performance of the RSF model was further evaluated. KIRP patients were stratified into low-risk and high-risk groups based on risk scores. Patients with high-risk scores had significantly shorter survival times (*p* < 0.001) compared with those in the low-risk group (Fig. 2D). Similar results were observed in the GSE2748 dataset (Fig. 2E). Furthermore, the AUC values in the TCGA dataset reached 0.983, 0.987, and 0.989 for predicting prognosis at 1, 3, and 5 years, respectively (Fig. 2F). The AUC values in the GSE2748 dataset were 0.923, 0.722, and 0.784 (Fig. 2G). Therefore, selecting the RSF as the optimal prognostic model provides improved insight into the metabolic heterogeneity of KIRP patients.

### Association of MRS with clinical features

**Fig. 3:**
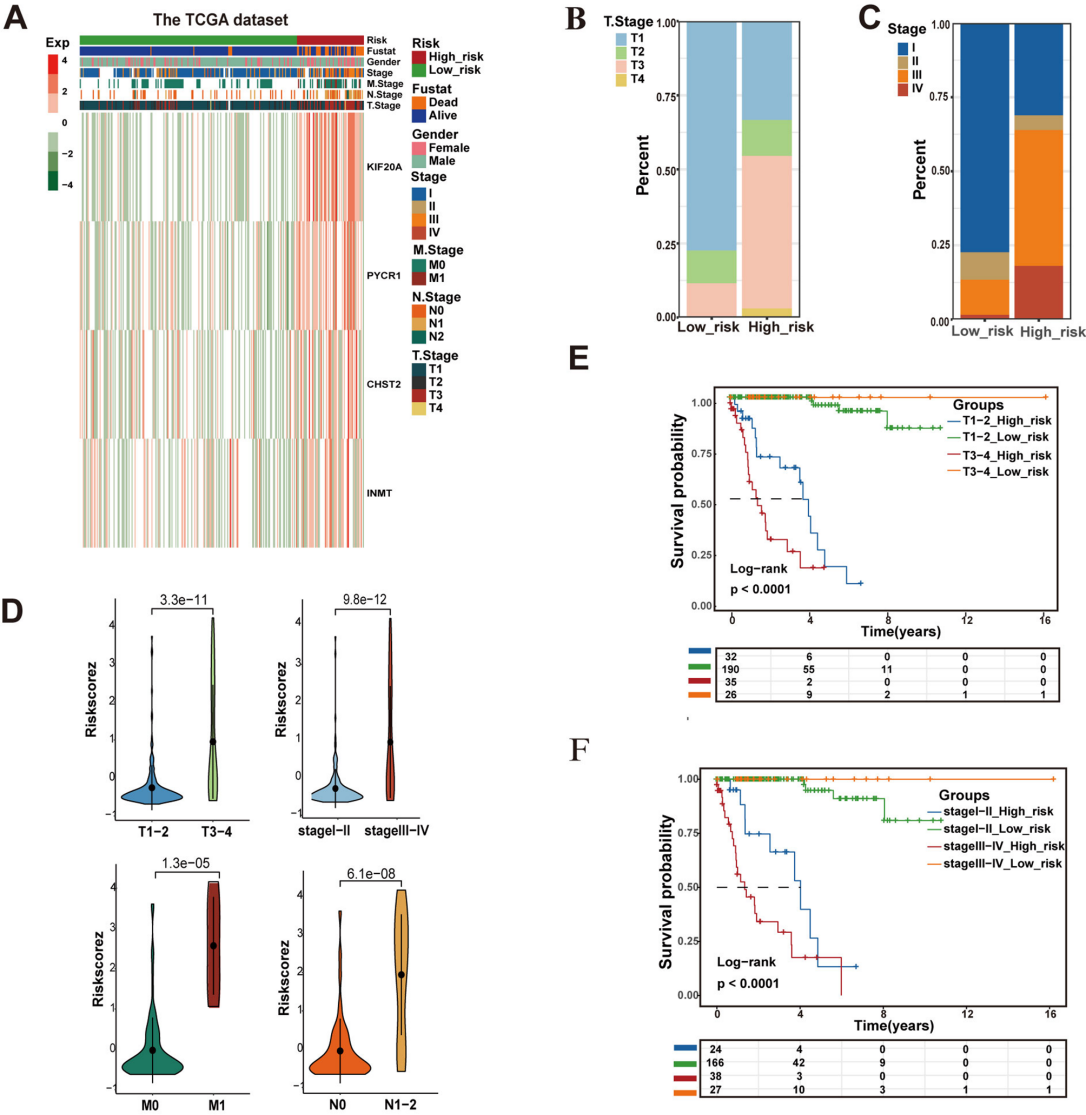
Exploring the Relationships between MRS and clinical features. (**A**) The distribution of clinical characteristics and the expression of RSF model genes. (**B**, **C**) The proportion of T stage (**B**) and pathological stage (**C**) in MRS risk subgroups. (**D**) The disparity in risk scores among patients categorized by T stage, M stage, N stage, and pathological stage. (**E**, **F**) Kaplan–Meier curves illustrating the performance within subgroups of KIRP patients, including T stage and pathological stage

To examine the association between the MRS and clinical features, we compared clinical characteristics between the high-risk and low-risk groups. Clear differences in the distribution of clinical characteristics were observed in the TCGA-KIRP cohort (Fig. 3A). Patients with advanced pathological stages or higher T stages were more likely to be categorized into the MRS high-risk group (Fig. 3B and C). Moreover, MRS risk scores were significantly higher (*p* < 0.001, *Wilcoxon test*) in patients with T3–4 stage, pathological stage III–IV, M1, and N1–2 compared with those with T1–2 stage, pathological stage I–II, M0, and N0 (Fig. 3D). When stratified by MRS risk scores, patients in the T3–4 high-risk group or T1–2 high-risk group had worse prognoses than those with low-risk scores (*p* < 0.001) (Fig. 3E). Similar results were observed when stratified by pathological stage (Fig. 3F). Taken together, the MRS shows significant potential for improving risk stratification in KIRP.

### Clinical prognosis and genomic variation burden of MRS

**Fig. 4:**
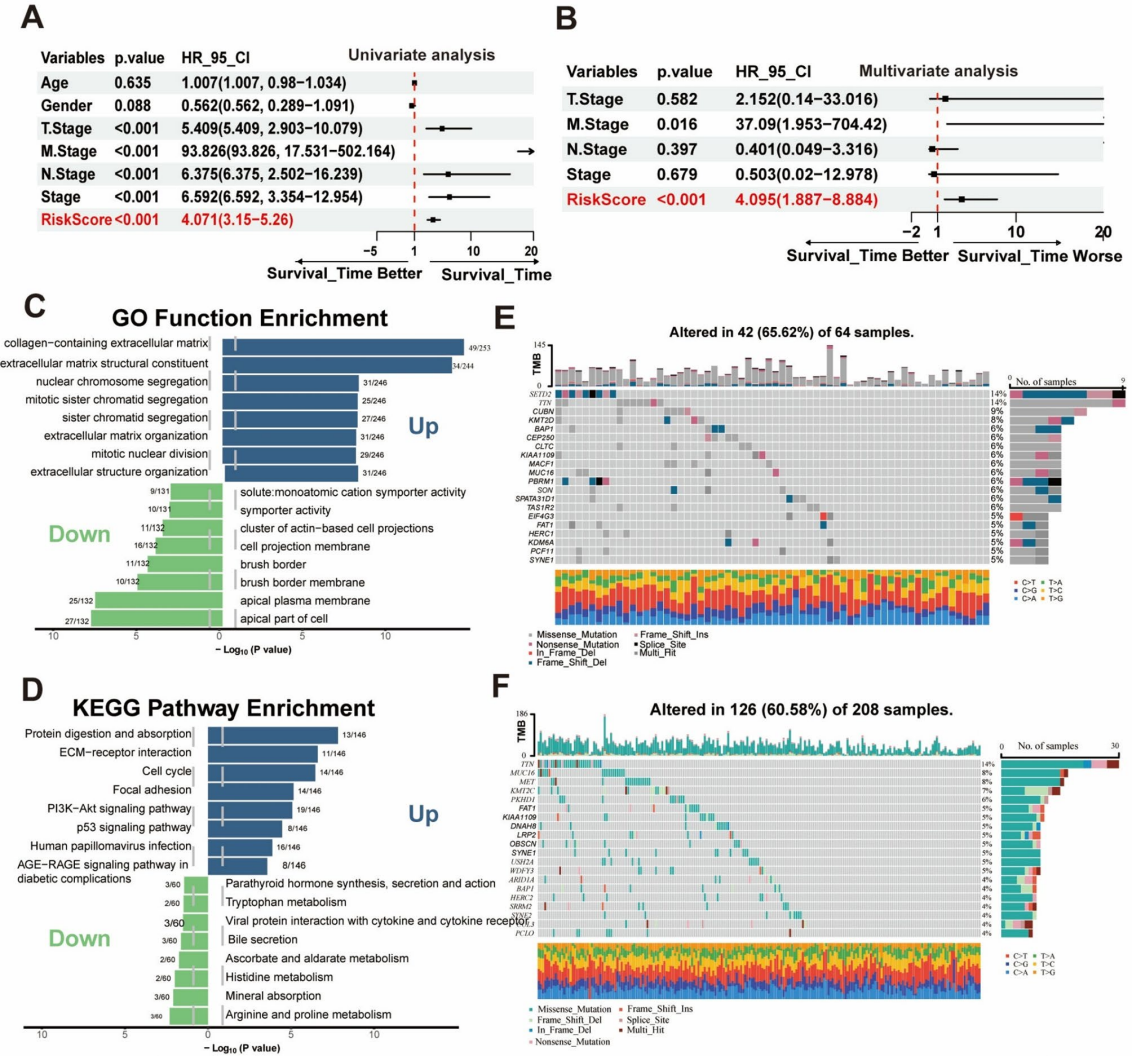
Functional enrichment analysis and genomic variation burden of MRS. (**A**, **B**) Univariate and multivariate analyses of clinical features and MRS for survival. (**C**) GO enrichment analysis between the high and the low-risk groups. (**D**) KEGG enrichment analysis. (**E**, **F**) The waterfall plot depicting somatic mutations in the high-risk group (**E**) and the low-risk group (**F**) of the TCGA KIRP cohort

We analyzed the distribution of tumor size and extent (T), regional lymph node involvement (N), and distant metastasis (M) stages within the patient cohorts and explored their associations with MRS risk scores. In addition, univariate and multivariate Cox regression analyses were performed to determine whether the MRS was an independent prognostic factor for KIRP. Univariate analysis indicated that the MRS risk score (HR: 4.071, 95% CI: 3.15–5.26), T stage, M stage, and N stage were risk factors for prognosis in KIRP (Fig. 4A). Moreover, in multivariate analysis, the MRS risk score also emerged as a poor prognostic factor (HR: 4.095, 95% CI: 1.887–8.884) for overall survival (Fig. 4B). However, stage and T stage were not statistically significant. We next performed GO and KEGG functional enrichment analyses using the results from the differential expression analysis. The high-MRS group was mainly enriched in several key biological pathways and components compared with the low-risk group. These included collagen-containing extracellular matrix, extracellular matrix structural constituent, nuclear chromosome, and mitotic sister chromatid segregation (Fig. 4C). In patients with high MRS scores, KEGG pathway analysis revealed elevated activity in pathways such as protein digestion and absorption, ECM–receptor interaction, cell cycle regulation, and the PI3K–Akt signaling pathway (Fig. 4D). Taken together, these results suggest a potential correlation between elevated MRS risk scores and poor prognosis.

Additionally, we investigated the genetic mutation landscape in KIRP and found no statistically significant difference in the total number of mutations between the high-risk and low-risk groups (*p* > 0.05, Supplementary Figure S1). We then examined the top 20 most frequently mutated genes within each risk group separately. Distinct mutation profiles were identified between the two MRS subgroups (Fig. 4E and F). The overall mutation rate in the high-risk group was 65.62%, exceeding that of the low-risk group, which was 60.58%. Notably, mutations in *SETD2*, an RNA polymerase II–associated histone methyltransferase known to promote metastasis in multiple cancers including renal cell carcinoma [21], were markedly more frequent in the high-risk group (14%) compared with the low-risk group (< 4%). These differences in mutated genes between the two MRS groups may contribute to distinct epigenetic landscapes and increased intra-tumor heterogeneity.

### Evaluating the RSF machine learning model using nomogram

**Fig. 5:**
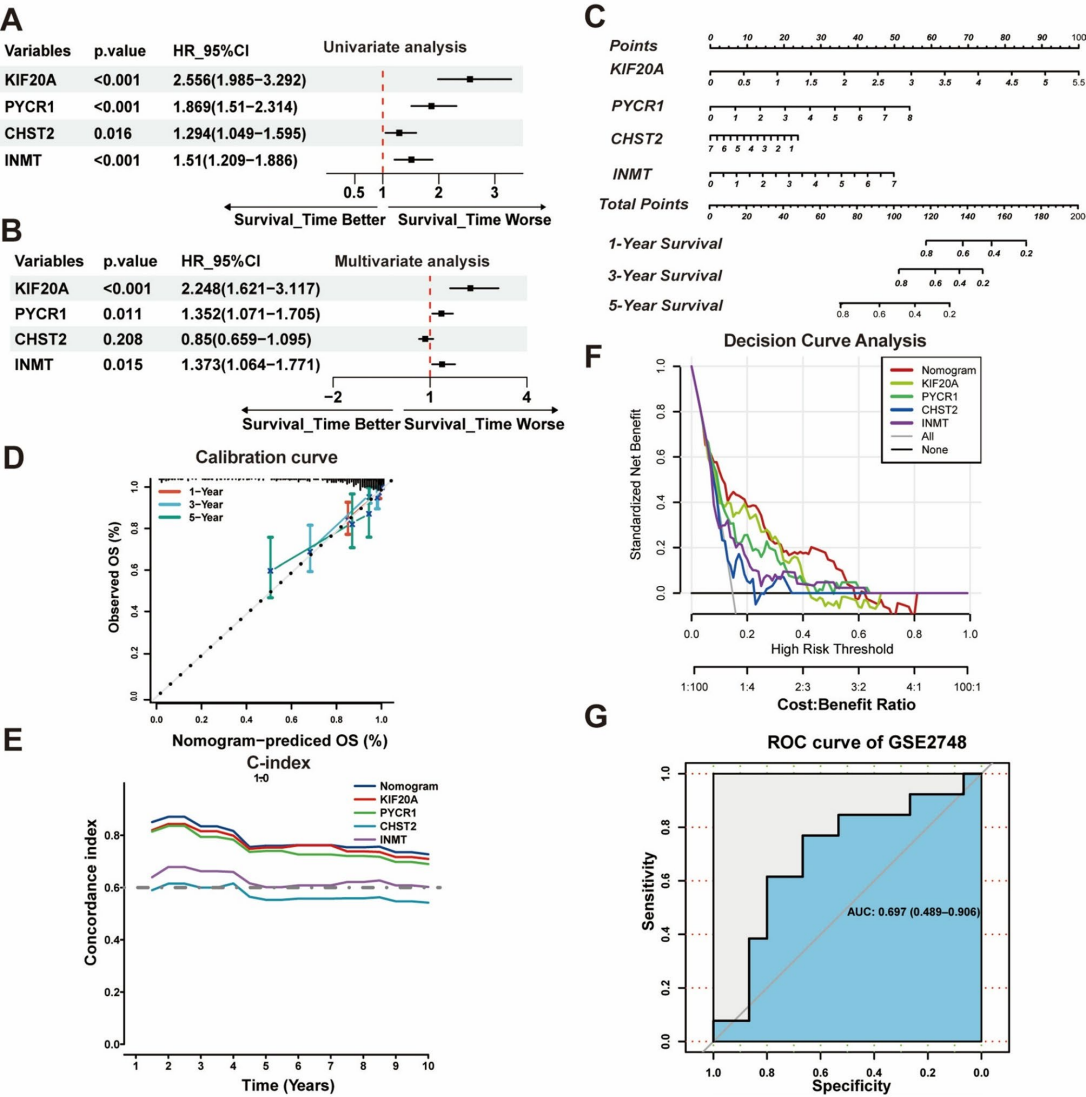
Establishment and validation of the nomogram. (**A**, **B**) Univariate (**A**) and multivariate analyses (**B**) the genes within MRS for the overall survive. (**C**) Construction of a nomogram to predict prognosis based on a 4-gene RSF model. (**D**) Calibration curve of the nomogram for 1, 3, and 5-year OS. (**E**) The *C-index* of the nomogram and four hub genes. (**F**) Decision curve analysis showing the net benefit of nomogram and other genes. (**G**) ROC analysis of showing the prediction performance of nomogram in validation cohort (*GSE2748*)

Univariate Cox regression analysis initially revealed a significant association between the four hub genes (*KIF20A*, *PYCR1*, *CHST2*, *INMT*) and poor survival outcomes (Fig. 5A), indicating their role as prognostic risk factors. In multivariate analysis, three of these genes (KIF20A, PYCR1, and INMT) remained independent prognostic risk factors, whereas CHST2 did not (Fig. 5B).

We then constructed a nomogram based on the four genes derived from the RSF machine learning model (Fig. 5C). The calibration curve demonstrated that the nomogram predictions were highly consistent with actual survival outcomes (Fig. 5D). The *C-index* of the line plots indicated robust predictive ability for overall survival across the 1- to 10-year timeframe (Fig. 5E). Decision curve analysis further confirmed favorable net clinical benefits (Fig. 5F), highlighting the potential utility of the nomogram for clinical decision-making. Finally, the model was validated in an external cohort (GSE2748), which consisted of a smaller set of KIRP patients. The AUC of the nomogram in this cohort reached 0.697, indicating moderate discriminatory ability (Fig. 5G).

### Immune cell infiltration analysis and drug sensitivity analysis

**Fig. 6:**
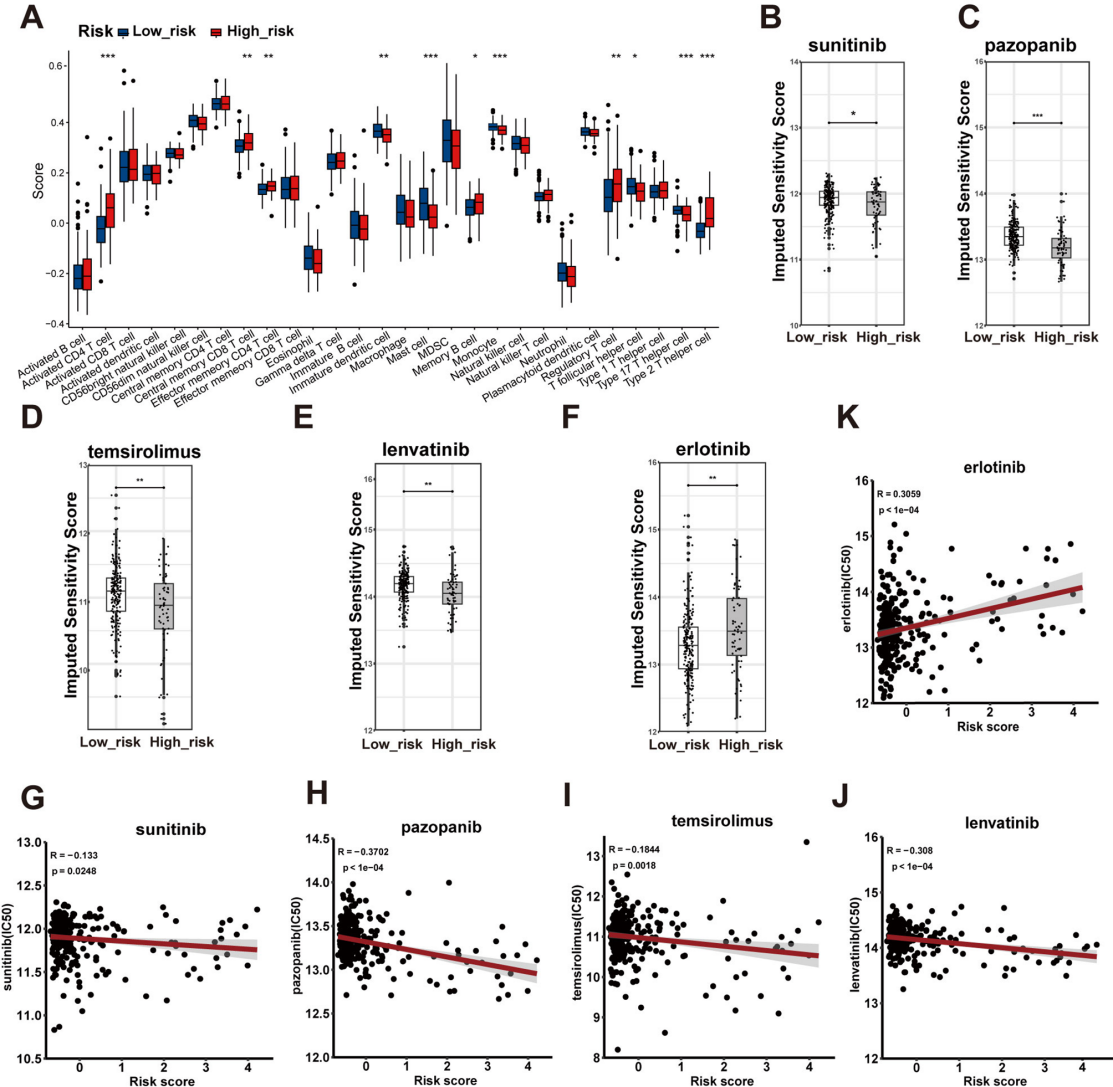
Immune cell infiltration and drug sensitivity. (**A**) The abundance of 28 immune cell infiltrations using the *ssGSEA* algorithm. (**B-F**) The drug sensitivity of sunitinib, pazopanib, temsirolimus, lenvatinib, and erlotinib. (**G-K**) The correlations between the MRS risk score and half-maximal inhibitory concentration (IC50) of sunitinib, pazopanib, temsirolimus, lenvatinib, and erlotinib

To analyze the immune cell infiltration landscape between the high-risk and low-risk groups, we examined 28 immune cell populations using the *ssGSEA* algorithm (Fig. 6A). Several immune cells were more abundant in the high-risk group, including activated CD4^+^ T cells, central memory CD8^+^ T cells, effector memory CD4^+^ T cells, memory B cells, regulatory T cells, and type 2 T helper cells. By contrast, infiltration levels of immature dendritic cells, mast cells, and follicular T helper cells were higher in the low-risk group.

TKIs and mTOR inhibitors are typically first-line drugs for the treatment of renal cell carcinoma. To improve drug selection, we evaluated the drug sensitivity of MRS risk subgroups to TKIs—sunitinib, pazopanib, lenvatinib, and erlotinib—and to the mTOR inhibitor temsirolimus (Fig. 6B and K). The IC50 of erlotinib was significantly lower in the low-risk group (Fig. 6F), indicating increased sensitivity to this drug. Moreover, there was a positive correlation between MRS risk scores and the IC50 of erlotinib (Fig. 6K). Conversely, patients with high MRS risk scores exhibited lower IC50 values for sunitinib, pazopanib, lenvatinib, and temsirolimus, suggesting greater sensitivity. These results indicate that patients in the high-risk group may respond more favorably to sunitinib, pazopanib, lenvatinib, and temsirolimus.

### Characteristics of the MRS at the single-cell level and validation of gene expression

**Fig. 7:**
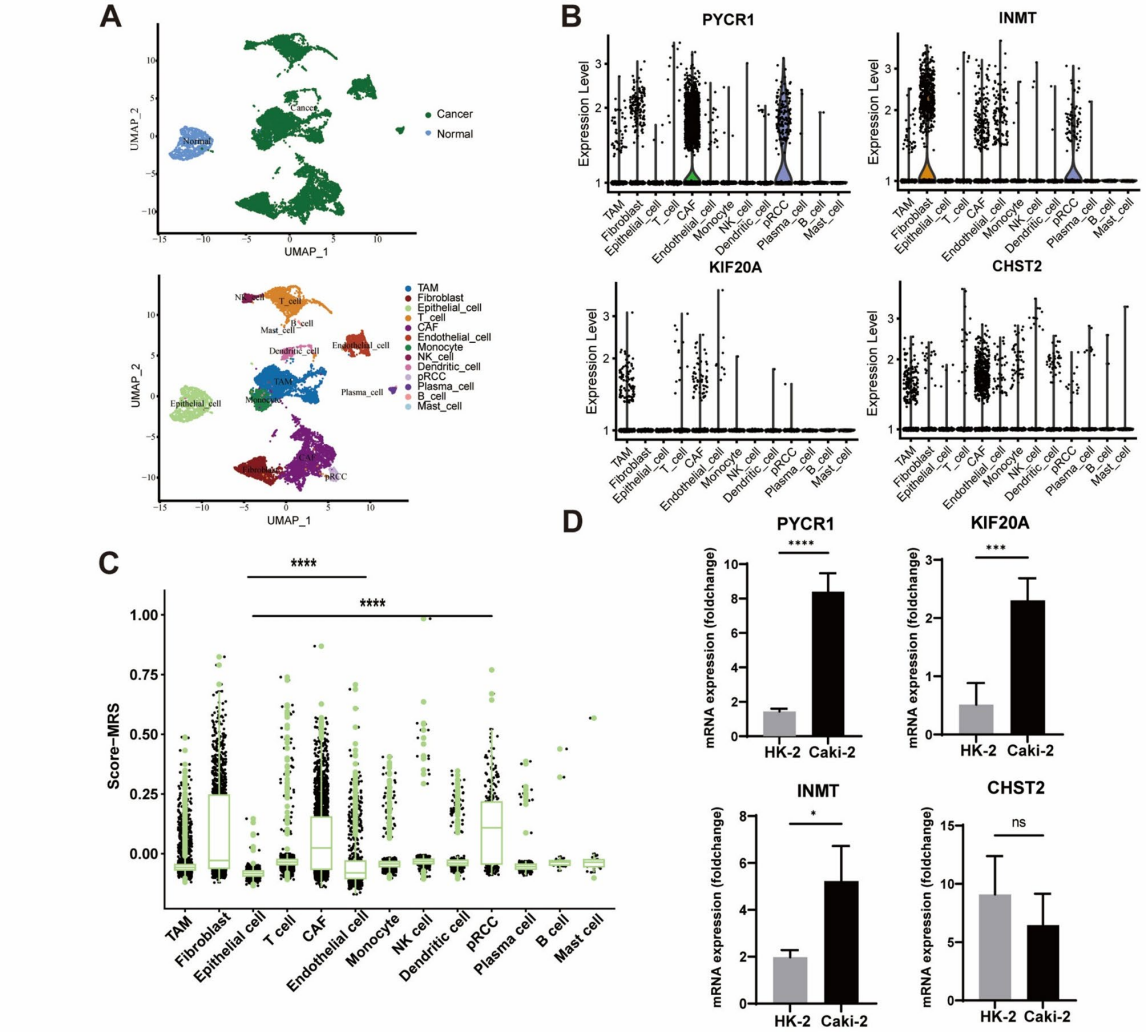
Evaluating the characteristic of MRS based on scRNA-seq. (**A**) Thirteen cell types were identified based on cell type-specific gene markers. (**B**) The expression levels of PYCR1, INMT, and KIF20A across various cell types. (**C**) Validation of the expression of PYCR1, INMT, and KIF20A in a normal kidney cell line (HK-2) and a cancer cell line (Caki-2) by qPCR. (**D**) The distribution of the MRS score across different cell types. (^*^p < 0.05, ^**^ *p* < 0.01, ^***^ *p* < 0.001, and ^****^ *p* < 0.0001)

To examine the transcriptomic characteristics of the MRS across renal cell types, we analyzed the scRNA-seq dataset *GSE152938*, which included data from one patient with KIRP and one healthy control (Fig. 7A). Using known marker genes, 13 clusters were manually identified (Fig. 7A), including tumor-associated macrophages (TAMs), fibroblasts, epithelial cells, T cells, cancer-associated fibroblasts (CAFs), endothelial cells, monocytes, NK cells, dendritic cells, papillary renal cell carcinoma cells (pRCCs), plasma cells, B cells, and mast cells [13]. The four MRS-related genes showed distinct expression patterns across different cell types. PYCR1 and INMT were predominantly expressed in CAFs, pRCCs, and fibroblasts (Fig. 7B). KIF20A was highly expressed in TAMs, with lower expression in epithelial cells. CHST2 displayed high expression in both CAFs and TAMs. Furthermore, we used the *ssGSEA* function to assess MRS activity and observed significantly higher levels in CAFs and pRCCs compared with other cell types, particularly epithelial cells (Fig. 7C**).** This finding is consistent with our bulk-RNA results.

**Fig. 8:**
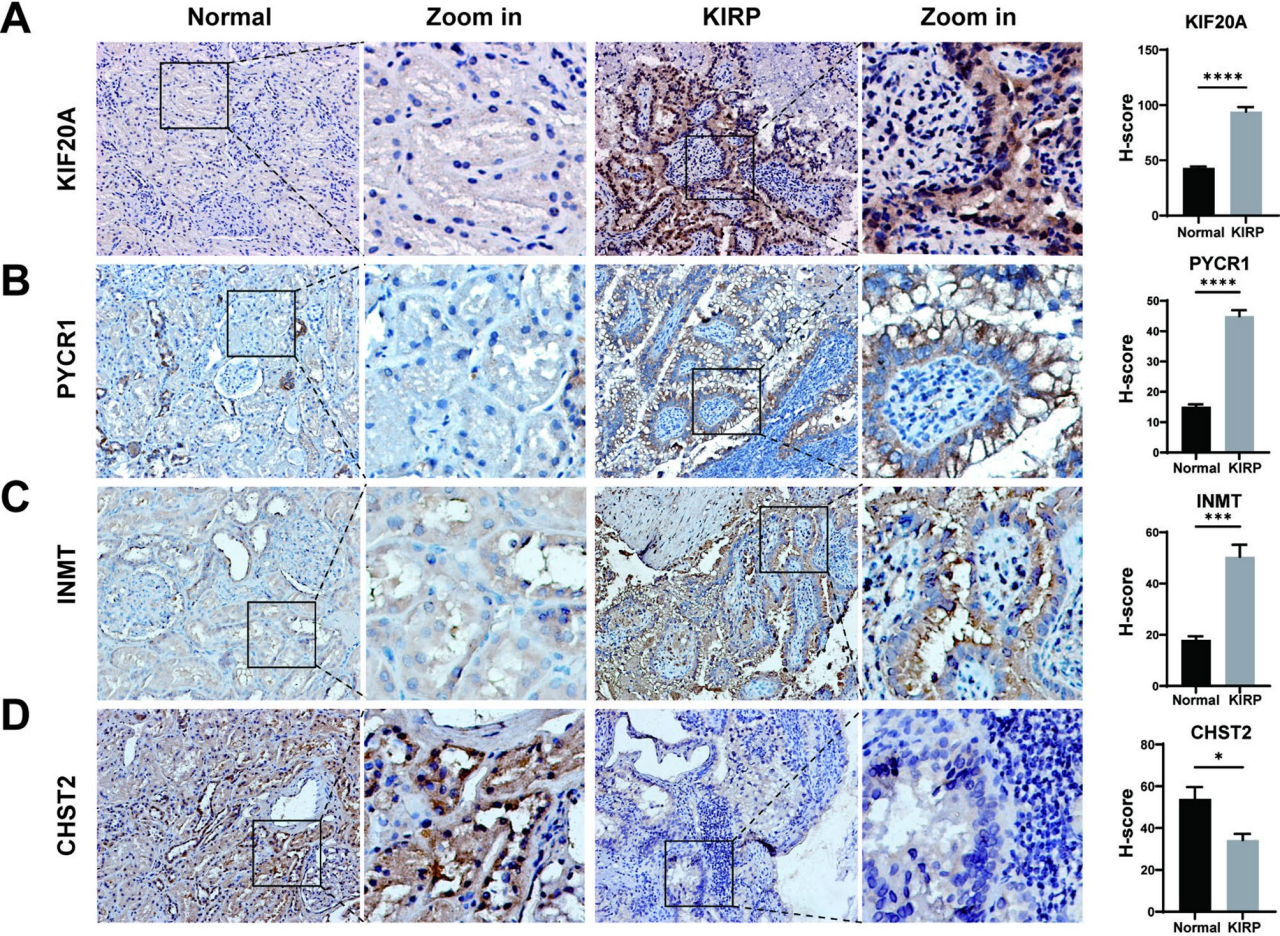
The immunohistochemical staining between normal renal tissues with KIRP. Representative IHC images show the protein expression patterns of (**A**). KIF20A(×200), (**B**) PYCR1(×200), (**C**) INMT(×200), and (**D**) CHST2(×200). (^*^p < 0.05, ^**^p < 0.01, ^***^p < 0.001, and ^****^p < 0.0001)

We next evaluated the expression of the four genes using qPCR and IHC staining. Our results showed that *PYCR1*, *INMT*, *and KIF20A* were significantly upregulated in tumor cells (Fig. 7D). Consistently, IHC staining of KIRP and normal renal tissues revealed similar expression patterns for these three genes (Fig. 8A-C). In contrast, *CHST2* mRNA levels did not differ significantly, possibly because this gene is predominantly expressed in CAFs and TAMs (Fig. 7B). At the protein level, CHST2 expression was markedly higher in normal renal tissues than in KIRP samples (Fig. 8D).

## Discussion

Renal cell carcinoma is increasingly recognized as a disease driven by metabolic alterations [10, 22]. Such metabolic reprogramming involves multiple genetic variants and dysregulated pathways, resulting in marked heterogeneity and complexity [23, 24]. Identifying metabolic characteristics in tumor tissues from a holistic perspective is crucial for improving patient management and treatment. However, the role of metabolic alterations in KIRP remains incompletely understood. In this study, we applied differential expression analysis and WGCNA to investigate the expression profiles of metabolism-related genes and elucidate their impact on the prognosis of KIRP. Furthermore, we developed 90 machine learning model combinations using 16 metabolism-related genes associated with survival outcomes and tumor severity, thereby demonstrating a novel and robust approach [16]. The RSF-based model achieved the best predictive performance (average *C-index*: 0.852). In parallel, a nomogram was constructed using four genes—*KIF20A*, *PYCR1*, *CHST2*, and *INMT*—which showed strong prognostic accuracy in the TCGA-KIRP cohort, highlighting its clinical utility. However, predictive performance was less pronounced in the *GSE2748* validation cohort (*C-index*: 0.735), likely due to the limited sample size. Overall, the prognostic efficacy of the MRS for KIRP was satisfactory.

Prior studies have revealed that more than 17 mutated genes were involved in metabolism, leading to altered metabolic patterns in renal cell carcinoma [8]. Therefore, we hypothesized that metabolic alterations are associated with clinical characteristics, including prognosis. Patients were classified into two risk groups according to the MRS. KIRP patients in the high-risk group exhibited more advanced clinical characteristics, including higher T stage, N stage, M stage, and pathological stage. When combined with survival outcomes, these differences became even more pronounced. The accumulation of genetic mutations drives the initiation and progression of carcinoma [25]. Although no statistically significant difference was observed in the total number of mutations between the low-risk and high-risk groups, distinct mutated genes were identified within each group. These unique mutation profiles may contribute to the observed differences in prognosis and metabolic patterns. Notably, we identified a high-frequency mutation in SETD2 in the high-risk group. SETD2, a histone methyltransferase, is recognized as a critical tumor suppressor in renal cancer [26]. Similar tumor-suppressive roles of SETD2 have also been reported in other cancer types [27, 28]. For instance, Yang et al. demonstrated that SETD2 inhibits the growth and metastasis of lung adenocarcinoma via STAT1–IL-8 signaling [27]. A recent study further revealed that SETD2 deficiency in renal tissues alters the metabolic microenvironment by affecting glycolysis, lipid metabolism, and sphingomyelin biosynthesis, thereby contributing to the development of clear cell renal cell carcinoma [9]. Therefore, mutation or loss of SETD2 function may significantly influence metabolism and prognosis in KIRP, potentially serving as a high-risk factor for disease progression. However, the specific mechanisms by which SETD2 exerts these effects require further experimental validation.

Metabolic alterations in the tumor microenvironment regulate the activation, differentiation, and function of immune cells, thereby shaping the tumor immune response [29]. In addition, the composition and phenotypic states of infiltrating immune cells significantly influence patient prognosis [30]. In this study, we observed differences in immune cell infiltration based on MRS risk classification. In the low-risk group, patients exhibited lower levels of immune cells that promote tumor growth, including type 2 T helper cells, regulatory T cells, and B cells. Moreover, levels of T follicular helper cells were reduced in the high-risk group, which was associated with better prognosis. These findings may partially explain why KIRP patients with high-risk scores were linked to poorer clinical outcomes. Notably, high levels of CD4^+^ T-cell and CD8^+^ T-cell infiltration did not confer significant survival benefits to patients with high MRS scores, underscoring the need for further investigation. Finally, MRS-based risk stratification may help guide clinical dosing strategies in KIRP. Patients in the high-risk group exhibited greater sensitivity to erlotinib, whereas those with low MRS scores were more likely to benefit from sunitinib, pazopanib, lenvatinib, and temsirolimus.

According to our findings, *PYCR1*, *KIF20A*, and *INMT* emerged as independent prognostic risk factors in KIRP. The scRNA-seq analysis showed that pRCC cells and CAFs exhibited high expression of *PYCR1*. A recent study reported that *PYCR1*, a key enzyme in proline biosynthesis, promotes the production of a collagen-rich extracellular matrix, thereby enhancing tumor growth and metastasis in breast cancer [31]. Consistently, our functional enrichment analysis also revealed similar findings, including the upregulation of collagen-containing extracellular matrix and ECM–receptor interaction pathways. Another study demonstrated that elevated *PYCR1* expression is associated with reduced immune cell infiltration and resistance to TKIs or immune checkpoint inhibitors [32]. Hence, *PYCR1* emerges as a promising antitumor target for KIRP patients. KIF20A is a member of the kinesin-6 family and is implicated in the progression of multiple tumors. Its upregulation promotes pyruvate and lactate production, and its regulation of metabolic processes contributes to colorectal cancer progression [33]. *KIF20A* also influences lipid metabolism and antioxidant defenses, thereby affecting resistance to oxidative stress and drug treatment in lung adenocarcinoma [34]. Similarly, suppression of KIF20A has been shown to inhibit invasion and infiltration in renal cancer [35]. Whether *KIF20A* promotes tumor progression in KIRP through metabolism-related pathways, however, requires further investigation. In addition, *INMT* is involved in the metabolism of tryptophan and selenium, catalyzing N-methylation reactions. *Zhong et al.* reported that silencing *INMT* expression reduced the castration resistance of prostate cancer through effects on methaneseleninic acid and selenocysteine hydrochloride [36]. Our study revealed that elevated INMT expression is associated with unfavorable clinical outcomes, indicating its potential as a therapeutic target. High expression of CHST2 promoted the migration and metastasis of breast cancer cells [37]. Interestingly, our results indicated that *CHST2* mRNA levels did not differ significantly, likely because this gene is predominantly expressed in non-tumor cells. Although the reduction of CHST2 protein may be regulated at the post-transcriptional level, upregulated CHST2 expression has also been suggested as a potential risk factor for KIRP. Our functional enrichment analysis revealed that KIRP cases with high MRS scores displayed enhanced activity in the PI3K–AKT signaling pathway. Notably, activation of the PI3K–AKT–mTOR pathway in renal cell carcinoma is a key driver of metabolic reprogramming and tumor progression [10]. This enhanced pathway activity may contribute to poorer survival outcomes in patients with high MRS scores.

While we performed a comprehensive analysis of the molecular characteristics of metabolism in KIRP, along with experimental validation, several limitations should be acknowledged. First, all datasets were obtained from public databases. The limited sample size of both the validation cohort and the scRNA-seq cohort constrained the predictive performance of the MRS. Second, the four genes comprising the MRS appear to play critical roles in shaping the altered metabolic landscape of KIRP. However, additional experimental studies are warranted to elucidate their functional roles and underlying mechanisms.

## Conclusions

The biogenesis and progression of KIRP are partly driven by metabolic alterations. This study comprehensively analyzed the molecular characteristics of metabolism in KIRP, revealing significant metabolic heterogeneity. The MRS exhibited significant clinical implications, including its strong association with prognosis, pathological stage. Additionally, a risk model based on the MRS was constructed for survival prediction. In conclusion, our findings provided novel insights into KIRP risk stratification, prognostic assessment, and therapeutic guidance.

## Supplementary Information

Below is the link to the electronic supplementary material.

Supplementary Material 1

## Data Availability

No datasets were generated or analysed during the current study.
