# Supplementary material for: Metabolic heterogeneity and survival outcomes in papillary renal cell carcinoma: insights from multi-datasets and machine learning analyses
Source: Hereditas. 2025 Sep 26;162:190. doi: 10.1186/s41065-025-00571-9 (PMC12465287; doi:10.1186/s41065-025-00571-9)

**Supplementary**

**Metabolic Heterogeneity and Survival Outcomes in Kidney Renal Papillary Cell Carcinoma: Insights from Multi-Datasets and Machine Learning**

**Supplementary Table S1**. The primer sequences for qPCR in this study.

| **Gene** | **Primer sequence (5-3’)** |
| --- | --- |
| INMT | F: *CTTCATTGTGGCTCGCA* |
|  | R: *CCATTCCTCACCCCTCTC* |
| CHST2 | F: *CTTCCCCTCCCTCGTTTC* |
|  | R: *TCCACTGCCCTGTTTGG* |
| PYCR1 | F: *GACACCCCACAACAAGGAG* |
|  | R: *GCGCAGGACACCACAAT* |
| KIF20A | F: *GGTTCACCTTTTCCCAGAT* |
|  | R: *GAGAATCCCTCCATCCTTG* |

**Supplementary Figure S1**. Comparison of the total number of mutations between the two MRS subgroups.


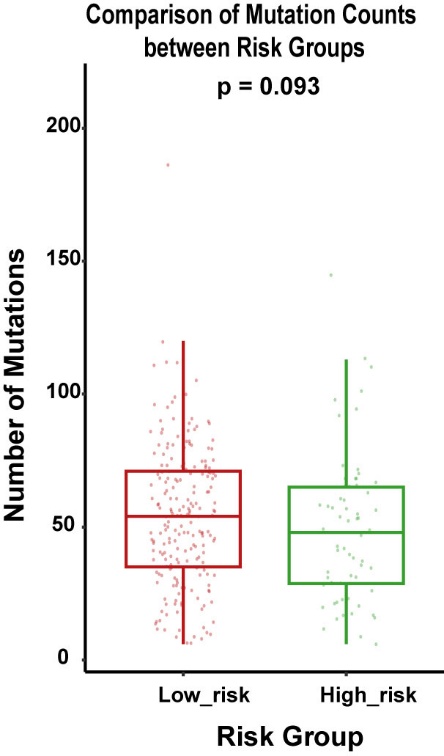

Supplement: Supplementary file 1 — Supplementary Material 1 [file 41065_2025_571_MOESM1_ESM.docx]
